# Integrated multi-level omics profiling of disulfidptosis identifis SPAG4 as an innovative immunotherapeutic target in glioblastoma

**DOI:** 10.3389/fimmu.2024.1462064

**Published:** 2024-10-30

**Authors:** Shenbo Chen, Man Zeng, Taixue Chen, Hui Ding, JiaHan Lin, Fuyue Ye, Ran Wu, Liangwang Yang, Kun Yang

**Affiliations:** ^1^ Department of Neurosurgery, The First Affiliated Hospital of Hainan Medical University, Haikou, Hainan, China; ^2^ Department of Geriatrics Center, The First Affiliated Hospital of Hainan Medical University, Haikou, Hainan, China

**Keywords:** glioblastoma, disulfidptosis, tumor immune microenvironment, prognostic model, SPAG4

## Abstract

**Objective:**

To investigate the association between disulfidptosis-related genes (DFRGs) and patient prognosis, while concurrently identifying potential therapeutic targets in glioblastoma (GBM).

**Methods:**

We retrieved RNA sequencing data and clinical characteristics of GBM patients from the TCGA database. We found there was a total of 6 distinct clusters in GBM, which was identified by the t-SNE and UMAP dimension reduction analysis. Prognostically significant genes in GBM were identified using the limma package, coupled with univariate Cox regression analysis. Machine learning algorithms were then applied to identify central genes. The CIBERSORT algorithm was utilized to assess the immunological landscape across different GBM subtypes. *In vitro* and *in vivo* experiments were conducted to investigate the role of SPAG4 in regulating the proliferation, invasion of GBM, and its effects within the immune microenvironment.

**Results:**

23 genes, termed DFRGs, were successfully identified, demonstrating substantial potential for establishing a prognostic model for GBM. Single cell analysis revealed a significant correlation between DFRGs and the progression of GBM. Utilizing individual risk scores derived from this model enabled the stratification of patients into two distinct risk groups, revealing significant variations in immune infiltration patterns and responses to immunotherapy. Utilizing the random survival forests algorithm, SPAG4 was identified as the gene with the highest prognostic significance within our model. *In vitro* studies have elucidated SPAG4’s significant role in GBM pathogenesis, potentially through the regulation of fatty acid metabolism pathways. Our *in vivo* investigations using a subcutaneous xenograft model have confirmed SPAG4’s influence on tumor growth and its capacity to modulate the immune microenvironment. Advanced research hints that SPAG4 might achieve immune evasion by increasing CD47 expression, consequently reducing phagocytosis.

**Conclusions:**

These findings highlight SPAG4 as a potential GBM therapeutic target and emphasize the complexity of the immune microenvironment in GBM progression.

## 1 Introduction

Glioma stands as the predominant primary malignant neoplasm within the central nervous system (CNS), comprising 47.7% of the total malignancies occurring in the CNS (1). Glioma is categorized into lower-grade gliomas (LGG, WHO grade I and II) and glioblastoma (GBM, WHO grade III and IV). Patients with glioblastoma generally experience a less favorable prognosis compared to those with lower-grade gliomas (2). The median survival period for glioblastoma (GBM) patients typically stands at 12 months post-surgery and radiotherapy. In contrast, the survival duration for lower-grade glioma (LGG) patients can vary significantly, spanning from 1 to 15 years (3). Therefore, it is of paramount importance to elucidate the molecular mechanisms that underlie the progression of GBM.

In 2023, Liu et al. (4) proposed a novel form of cell death termed dual sulfur death. In their investigation, they observed that under conditions of glucose starvation, cells with elevated expression of solute carrier family 7 member A11 (SLC7A11) led to the continuous accumulation of intracellular disulfides. This subsequently induced a stress response resulting in increased disulfide bond content in the actin cytoskeleton, a component of the cell structure. The heightened disulfide bonds prompted contraction of actin filaments, ultimately disrupting the cytoskeletal architecture and leading to cell death (disulfidptosis). This new form of cell death differs from well-known mechanisms such as apoptosis, autophagy, and necroptosis. An increasing body of research suggests that disulfidptosis plays a crucial role in the initiation and progression of tumors and may serve as a novel molecular target (5, 6). However, as of now, the specific role of disulfidptosis in GBM remains unclear.

In this study, our objective was to comprehensively investigate the role of disulfidptosis-related genes (DFRG) in the immune microenvironment and prognosis of GBM. We stratified GBM patients into distinct risk groups based on the expression levels of disulfidptosis-related genes (DFRG) and their association with clinical outcomes. We developed and validated a prognostic model associated with DFRG, which demonstrated high accuracy in predicting prognosis and immune therapy response across various independent cohorts. SPAG4 emerged as the most crucial gene in the model. Mechanistically, SPAG4 was found to facilitate the transformation of macrophages into an M2 phenotype, thereby promoting the proliferation and invasion of GBM. These findings provide potential therapeutic targets for the treatment of GBM.

## 2 Materials and methods

### 2.1 Ethics statement

This study has been approved by the Ethics Committee of The First Affiliated Hospital of Hainan Medical University. The acquisition of tumor and adjacent normal tissues for GBM patients has been agreed by the patients themselves, and the informed consent has been signed. The specimen tissues of these clinical patients were used for subsequent Western blot and immunohistochemical analysis.

### 2.2 GBM data acquisition

In this study, the TCGA-GBM cohort comprising 374 glioblastoma (GBM) samples and 50 normal tissue samples was utilized. Gene expression profiles and clinical data, including TNM classification, age, gender, and overall survival, were obtained from the TCGA data portal (https://portal.gdc.cancer.gov/).

In addition, we acquired the GSE14520 dataset, consisting of 221 GBM samples, from the GEO database (https://www.ncbi.nlm.nih.gov/geo/), and the ICGC dataset (https://icgc.org/), which included 240 GBM samples. Only datasets with comprehensive clinical information were included in the analysis.

Furthermore, single-cell data was sourced from the Tumor Immune Single-Cell Hub (TISCH2; http://tisch.comp-genomics.org), an extensive online repository of single-cell RNA-seq data specifically focusing on the tumor microenvironment (TME) (1). Leveraging this resource, we systematically explored TME heterogeneity across diverse datasets and cell types.

### 2.3 DFRGs resource

A comprehensive set of 25 disulfidptosis-related genes (DFRGs) was acquired from the GeneCards repository.

### 2.4 Consensus clustering

For cluster analysis, the “ConsensusClusterPlus” package was utilized, employing the k-means algorithm. To identify genes exhibiting significant alterations across distinct clusters of disulfidptosis-related genes (DFRGs), differential expression analysis was performed using the “limma” software. Criteria for determining the significance of changes included a False Discovery Rate (FDR) < 0.05 and an absolute log2 fold change (|log2FC|) > 1.

### 2.5 Cell culture and cell lines

HEB cells, U87 cells, and U251 cells were cultured separately in DMEM high-glucose medium containing 10% FBS and 1% penicillin-streptomycin solution (dual antibiotics, P/S). MCF-10A cells were cultured in DMEM/F12, supplemented with 5% horse serum, 20 ng/mL epidermal growth factor (EGF), 0.5 μg/mL hydrocortisone, 10 μg/mL insulin, 1% non-essential amino acid solution (NEAA), and 1% penicillin-streptomycin solution (dual antibiotics, P/S). All cells were maintained in a 37°C, 5% CO_2_ incubator, with passaging conducted every 2-3 days. The culture medium was replaced twice a week.

### 2.6 Measurement of cellular reactive oxygen species levels

Following various treatments, cells were cultured for 3 days in a 37°C, 5% CO_2_ incubator. Staining was conducted according to the instructions provided by the cell proliferation assay kit. Images were captured and observed under a laser confocal microscope, and the proportion of DCFH-DA-stained positive cells was analyzed.

### 2.7 Western blot experiment

Total proteins were extracted using a lysis buffer. Subsequently, total proteins (40 μg/sample) were separated through 10% SDS-PAGE, running at 300 mA for 120 minutes. Following electrophoresis, proteins were transferred to a PVDF membrane, and the membrane was blocked with 5% milk at room temperature for 1 hour. Next, the membrane was incubated overnight at 4°C with the primary antibody, followed by a 2-hour incubation with the corresponding secondary antibody at room temperature. Finally, the expression levels of the target protein bands were analyzed. The following primary antibodies were utilized: anti-SPAG4 (1: 1000, CSB-PA989865), and anti-GAPDH (1: 5000, CSB-MA000071M1m) were purchased from Cusabio Biotech Co. Ltd, Hubei, China. Anti-CD47 (1: 2000, AF6423) was purchased from Beyotime Institute of Biotechnology, Nantong, China.

### 2.8 Cell scratch assay

Cell scratch assay was performed to assess cell migration. U87 and U251 cells from siNC group, siRNA1 group, and siRNA2 group were trypsinized, counted using a cell counting plate, and seeded into 6-well plates at a density of 2×10^5^ cells/well in serum-free medium. After cell fusion, scratches were made using a 200 μL pipette tip. Subsequently, the cells were washed twice with PBS, and the cell-free areas were photographed at 0 and 24 hours, respectively.

### 2.9 Transwell invasion assay

This experiment was employed to assess the invasive capabilities of U87 and U251 cells. Cells from siNC group, siRNA1 group, and siRNA2 group were starved for 24 hours and then seeded at a density of 3×10^4^ cells/well in the upper chamber of Transwell, which was pre-coated with Matrigel. The upper chamber was devoid of FBS, while the lower chamber contained DMEM with 10% FBS. After 24 hours, cells were fixed with 4% paraformaldehyde for 30 minutes, followed by staining with 0.1% crystal violet. Non-invasive cells in the upper chamber were gently removed with a cotton swab, and images were captured using a high-power inverted microscope.

### 2.10 Immunohistochemical staining

Tissue cores on the microarray were deparaffinized in xylene, followed by graded ethanol hydration. Antigen retrieval was achieved by microwave treatment in a citrate buffer solution. Subsequent immunohistochemical staining was performed according to the kit instructions, with the primary antibody of SPAG4 (CSB-PA989865, Cusabio Biotech) diluted to a working concentration of 1:400 and incubated overnight at 4°C.

### 2.11 Immunofluorescence staining

Tissue sections were deparaffinized in xylene, underwent graded ethanol hydration, and antigen retrieval was performed using microwave treatment in a citrate buffer solution. After room temperature blocking, the sections were incubated with the primary antibody overnight at 4°C. The next day, the sections were incubated with a mixture of goat anti-rabbit red fluorescence secondary antibody (ab150080, Abcam USA, working concentration 1:1000) and goat anti-mouse green fluorescence secondary antibody (ab150117, Abcam USA, working concentration 1:1000). After washing, the sections were counterstained with DAPI-containing anti-fluorescence quenching mounting medium, cover-slipped, stored in the dark at 4°C, and examined and imaged using an upright fluorescence microscope. The following primary antibodies were utilized: anti-induction of brown adipocytes 1 (Iba-1, 1: 500, GB15105-100), anti-inducible nitric oxide synthase (1: 500, iNOS GB115703-100), and anti-CD206 (1: 500, GB113497-100) were purchased from Servicebio, Wuhan, China.

### 2.12 Subcutaneous tumor xenograft in nude mice

U251 cells in logarithmic growth phase were centrifuged, counted, and based on the cell count, a portion of the cells was resuspended in DMEM to achieve a concentration of 2 × 10^6^ cells/100 μL. Nude mice were prepared by disinfecting the left shoulder with iodophor using a cotton ball. Using a syringe, 100 μL of cell suspension was injected into the left shoulder subcutaneously. After injection, gentle pressure was applied to stop bleeding. After approximately one week of normal housing, the tumors on the nude mice were measured using calipers. Once the tumors reached an approximate volume of 70 mm^3, mice were randomly divided into groups of 5, housed separately as control and intervention groups. The mice’s weight was recorded during each administration to monitor any changes after drug administration.

### 2.13 Statistical analysis

R version 4.2.3 software was employed for data processing, statistical analysis, and visualization. The optimal cut-off value was determined using the “survminer” R package, and Kaplan-Meier analysis was conducted using the survival program. Comparisons between two groups regarding continuous variables were performed using the Wilcoxon rank-sum test, while Spearman correlation analysis was employed to assess the interrelationships among continuous variables. Statistical significance was defined as P < 0.05 for all conducted statistical analyses.

## 3 Results

### 3.1 The mutation profile of DFRG in GBM

**Figure 1:**
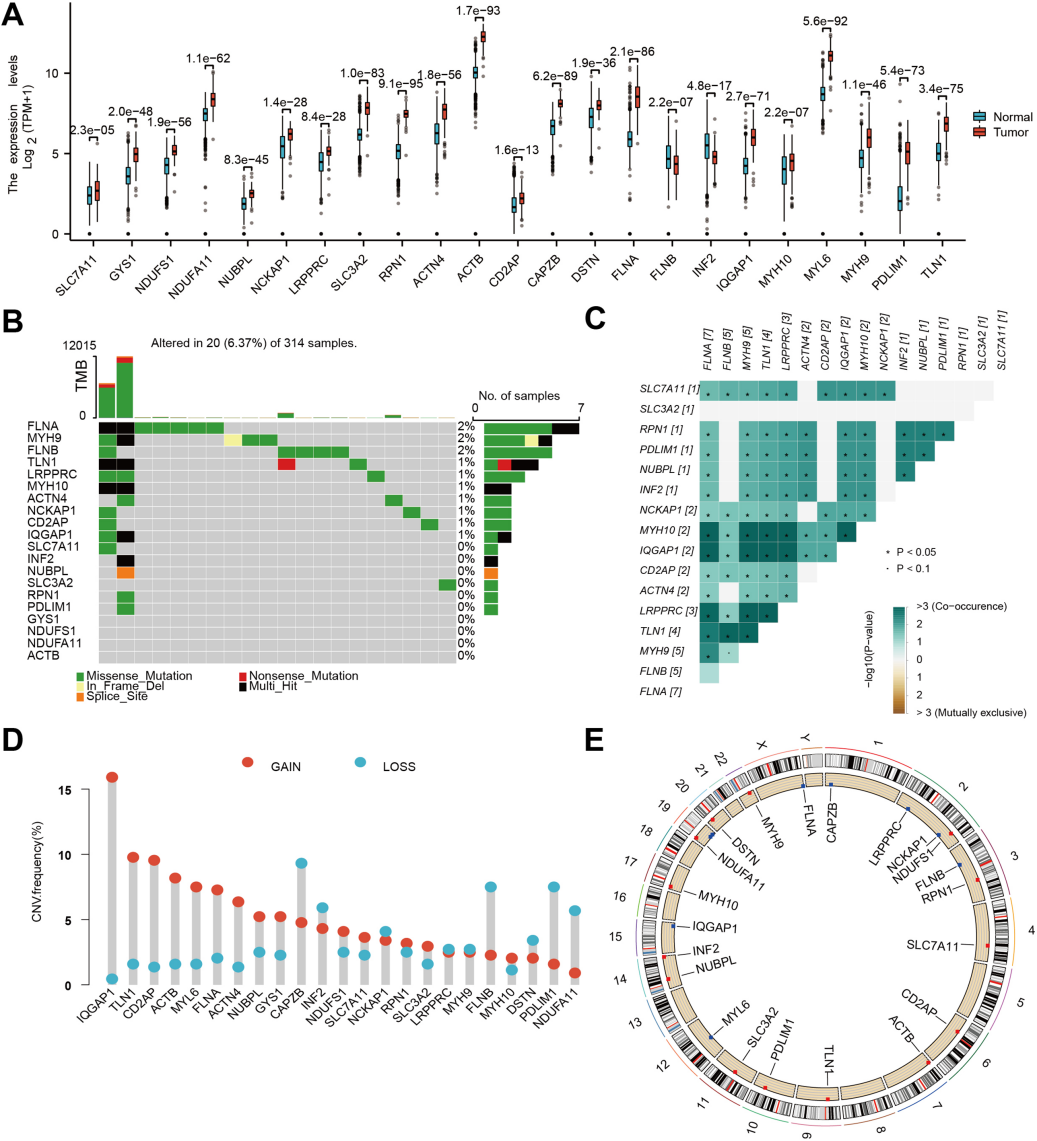
Genetic and transcriptional characteristics of DFRGs. **(A)** Gene expression levels from TCGA+GTEx V8 tissues. **(B)** Somatic mutation of DFRGs in the study population. **(C)** Gene alteration co-occurrence and mutual exclusivity analysis heatmap. **(D)** Frequency of gene copy number variations (gains and losses) in DFRGs. **(E)** Chromosome region of DFRGs.

In order to elucidate the molecular underpinnings of glioblastoma (GBM) and identify potential therapeutic targets, we initiated our investigation by examining the expression patterns of DFRGs within GBM, utilizing the comprehensive GTEx database. Utilizing the criteria of |logFC| > 1 and adjusted P.Value < 0.05, we identified a total of 217 upregulated and 149 downregulated differentially expressed genes, encompassing 23 DFRGs (**Supplementary Figure S1**). As depicted in **Figure 1A**, our comparative analysis of 23 DFRGs. Building upon these findings, we further explored the genetic stability of the 23 DFRGs. Mutational profiling was conducted to assess whether these genes harbored any oncogenic mutations. It showed that 19 of the DFRGs exhibited mutations, while 4 genes remained mutationally unaltered (**Figure 1B**). To gain insight into the cooperative or mutually exclusive relationships among these genes, we performed a gene alteration co-occurrence and mutual exclusivity analysis (**Figure 1C**). Gene copy number variations, including gains and losses, are known to significantly impact gene expression and contribute to cancer phenotypes, which is shown in **Figure 1D**. In order to contextualize the chromosomal localization of DFRGs and infer potential mechanisms of genomic instability, we generated a graphical representation of the chromosome regions harboring these genes. **Figure 1E** presents a genomic landscape of DFRGs, suggesting that their spatial distribution may have implications for GBM genetics and therapeutic targeting.

### 3.2 Expression of DFRG in the single-cell analysis within GBM

**Figure 2:**
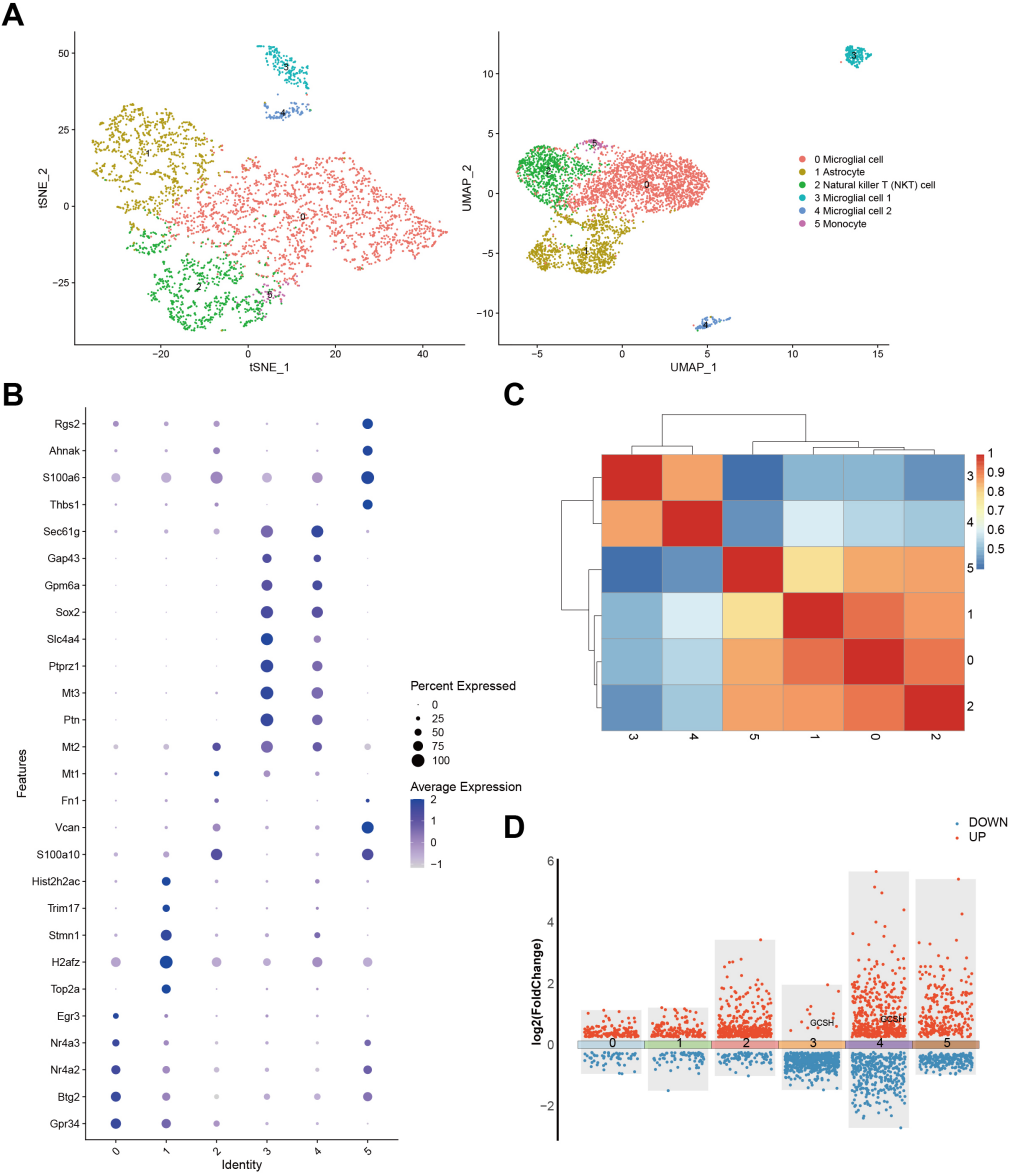
Expression of DFRG in the single-cell analysis and the data processing results of GSE241037. **(A)** The t-SNE analysis result of cells and the UMAP analysis plot. **(B)** A bubble diagram was generated to depict the expression identity of DFRG. **(C)** Correlation analysis was performed to examine the gene expression profiles among distinct cell types. **(D)** A differential expression gene map was constructed.

To understand the cellular context and functional significance of DFRGs in GBM, we compared their expression patterns within single-cell resolution GBM samples from the GSE241037 dataset. Our approach involved utilizing both t-SNE and UMAP analyses to visualize the cellular distribution (**Figure 2A**). Further exploration of DFRG expression was achieved through a bubble diagram representation presented in **Figure 2B**. **Figure 2C** illustrated the gene expression profiles and interrelationships among distinct cell types. **Figure 2D** showed a significant upregulation of GCSH in microglial cells, suggesting a correlation between disulfidptosis and tumor immune modulation.

### 3.3 Disulfidptosis subtypes compared through analyses

**Figure 3:**
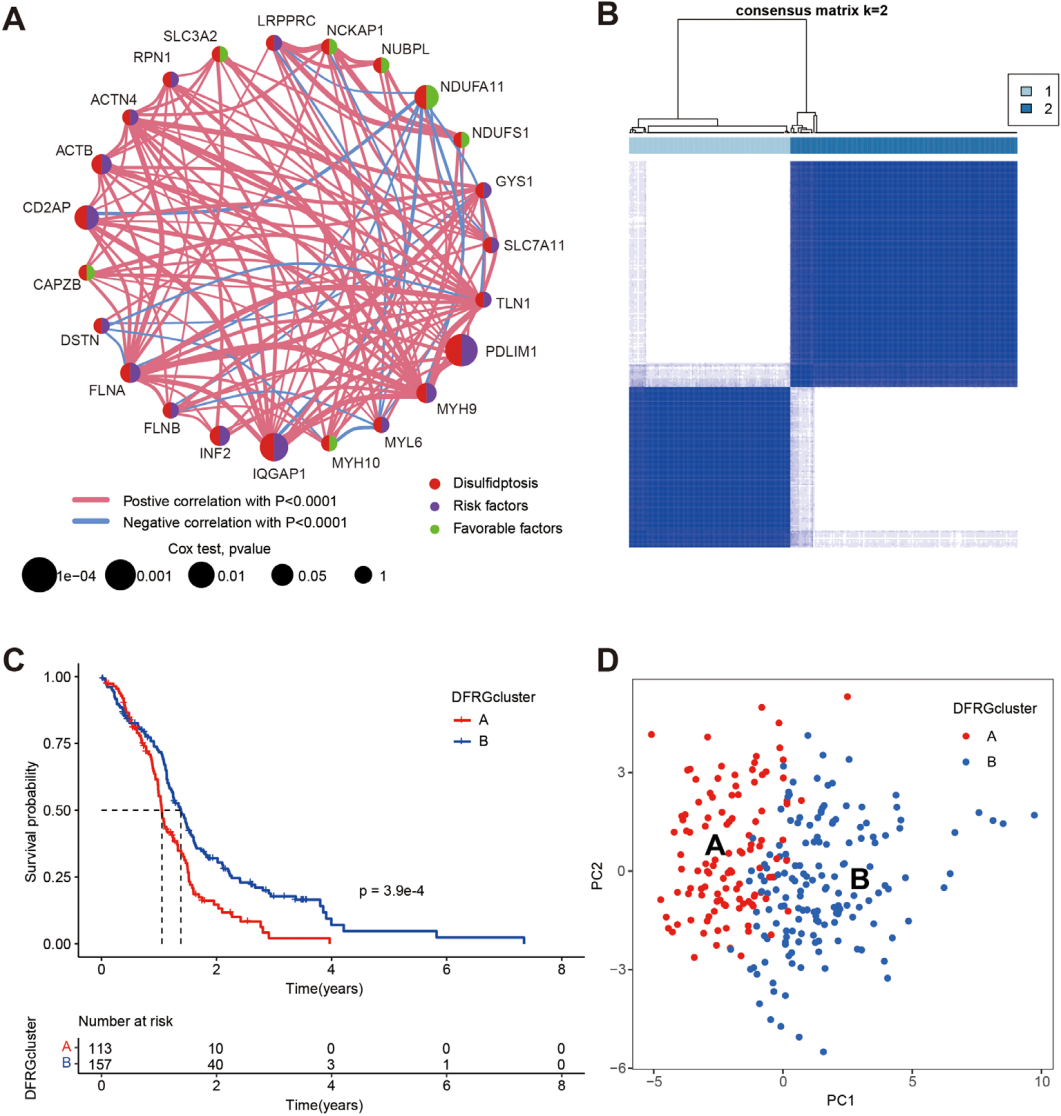
Identification of disulfidptosis subtypes and comparison of clinical outcomes between the two subtypes, data were from TCGA, GSE74187, and GSE83300. **(A)** Univariate Cox regression and correlation analysis between DFRGs. **(B)** Unsupervised clustering analysis of prognostic DFRGs. Consensus matrix heatmap defining two clusters (k = 2) and their correlation area. **(C)** Kaplan-Meier curves for OS of the two disulfidptosis subtypes (chi-square test, p = 3.9e-04). **(D)** The PCA analysis based on the prognostic DFRGs demonstrated that the patients in the different disulfidptosis subtypes were distributed in two directions.

To elucidate the complex interactions among DFRGs in GBM and their prognostic significance, we conducted a comprehensive analysis by integrating data from TCGA and GEO (GSE74187, and GSE83300), which is displayed in **Figure 3A**. Subsequently, to identify prognostic DFRGs, we performed an unsupervised clustering analysis, as depicted in **Figure 3B**, which resulted in a consensus matrix heatmap defining two distinct clusters (k = 2). The DFRG-related clusters, discerned through the aforementioned methodologies, exhibit notable prognostic relevance, as illustrated in **Figure 3C** with Kaplan-Meier curves depicting overall survival (OS).The comparison revealed a significant difference in survival rates between the two disulfidptosis clusters (chi-square test, p = 3.9e-04). To further explore the heterogeneity between the DFRG-related clusters, **Figure 3D** presents a principal component analysis (PCA). The result illustrates the distinct distribution patterns of patients, indicating that the identified DFRGs could be used to stratify GBM patients into subgroups with different clinical outcomes.

### 3.4 Cluster analysis outcomes for DFRG

**Figure 4:**
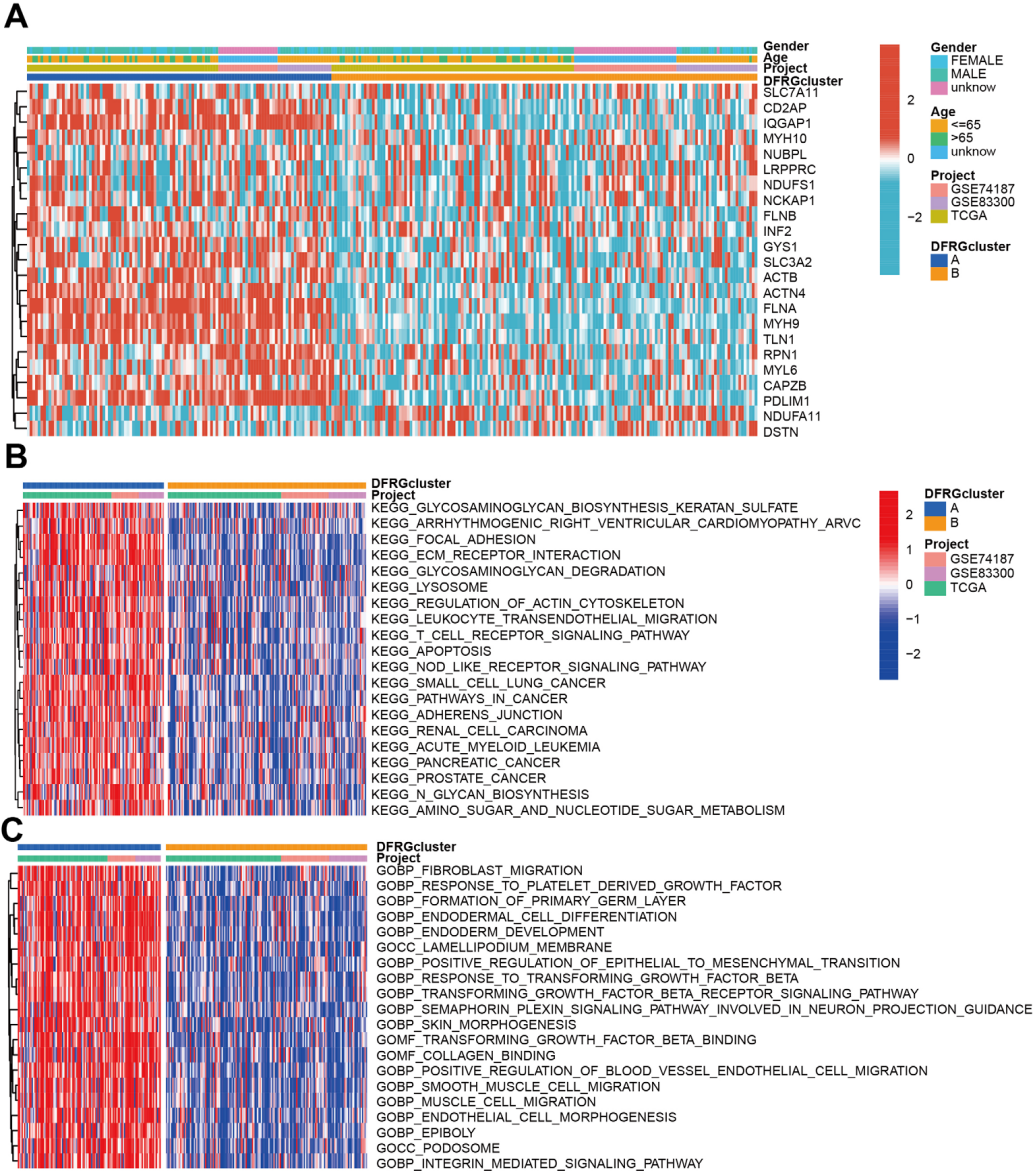
Comparison of clinical characteristics and KEGG and GO enrichment in each distinct cluster. **(A)** The heat map shows the differences in clinical features and DFRGs expression levels between DFRGcluster A and DFRGcluster B. **(B)** KEGG pathway enrichment by GSVA. **(C)** GO enrichment by GSVA.

To understand the clinical relevance and molecular mechanisms underlying the distinct subtypes of GBM defined by DFRGs, we compared the clinical characteristics and performed functional enrichment analyses between DFRGcluster A and DFRGcluster B. GSVA method was utilized to explore the enrichment of KEGG pathways and GO terms. As shown in **Figure 4A**, significant disparities were found in clinical features and expression levels of DFRGs between the two clusters. **Figure 4B** depicts the KEGG pathway enrichment analysis using GSVA, revealing distinct biological pathways that are overrepresented in each cluster. Similarly, **Figure 4C** showcases the GO enrichment analysis through GSVA, indicating the functional categories that are significantly enriched in each DFRG cluster.

### 3.5 Tumor-infiltrating immune cells and functional enrichment analysis results

**Figure 5:**
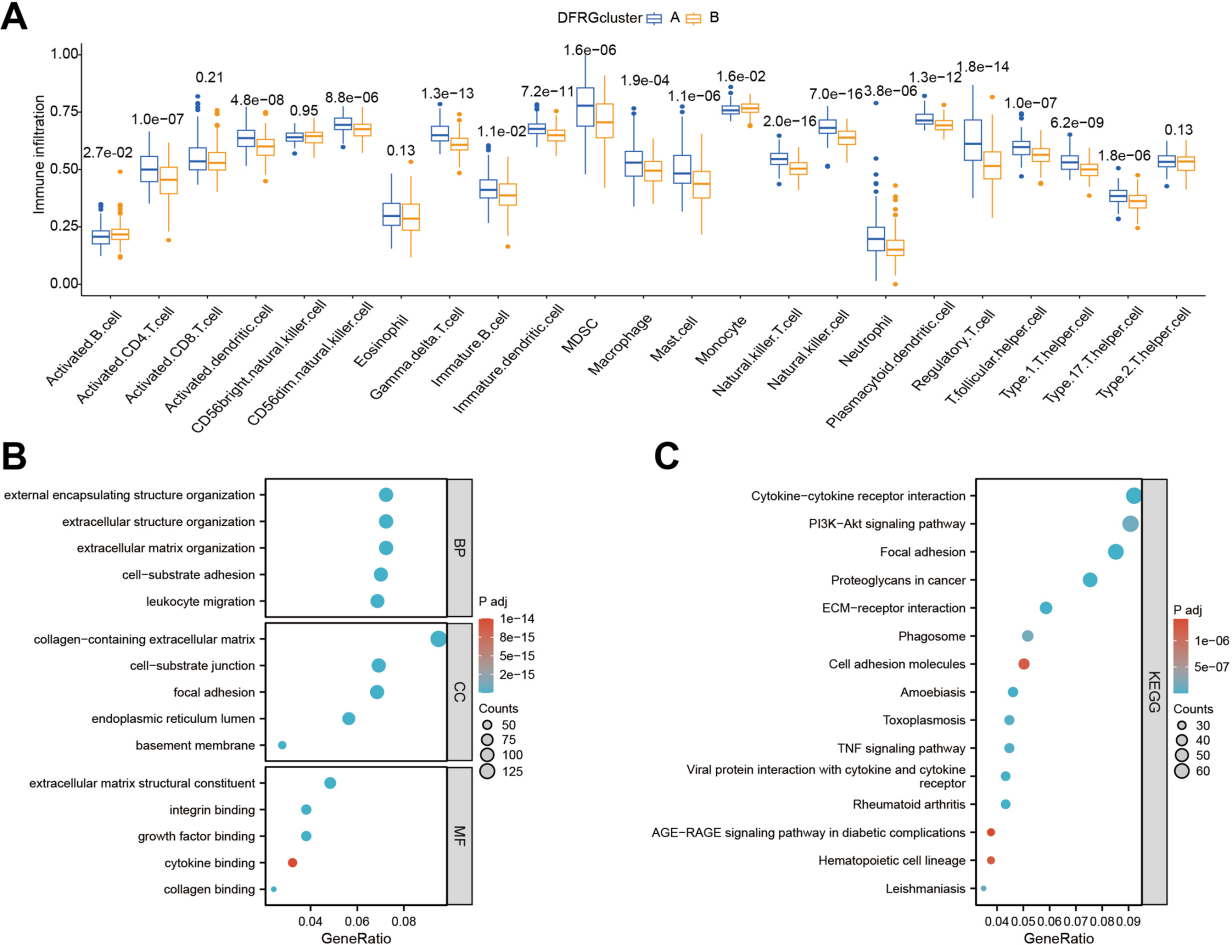
Comparative analysis of immune infiltration and pathway enrichment across different disulfidptosis clusters. **(A)** Comparison of tumor-infiltrating immune cell abundance between different disulfidptosis clusters. **(B)** Results of Gene Ontology (GO) enrichment analysis for differentially expressed genes in clustered groups. **(C)** Results of KEGG pathway enrichment analysis for differentially expressed genes in clustered groups.

To discern the immunological landscape and molecular pathways associated with different DFRG-related clusters in glioblastoma, we conducted a comparative analysis of tumor-infiltrating immune cell abundance and performed GO and KEGG pathway enrichment analyses. As shown in **Figure 5A**, certain immune cell types were found enriched in specific disulfidptosis clusters, suggesting a potential role in disease progression or response to therapy. In **Figures 5B, C**, the results of the GO and KEGG enrichment analyses for DFRGs between the two clusters were provided.

### 3.6 Clinical and pathological characteristics among patients of distinct subtypes

**Figure 6:**
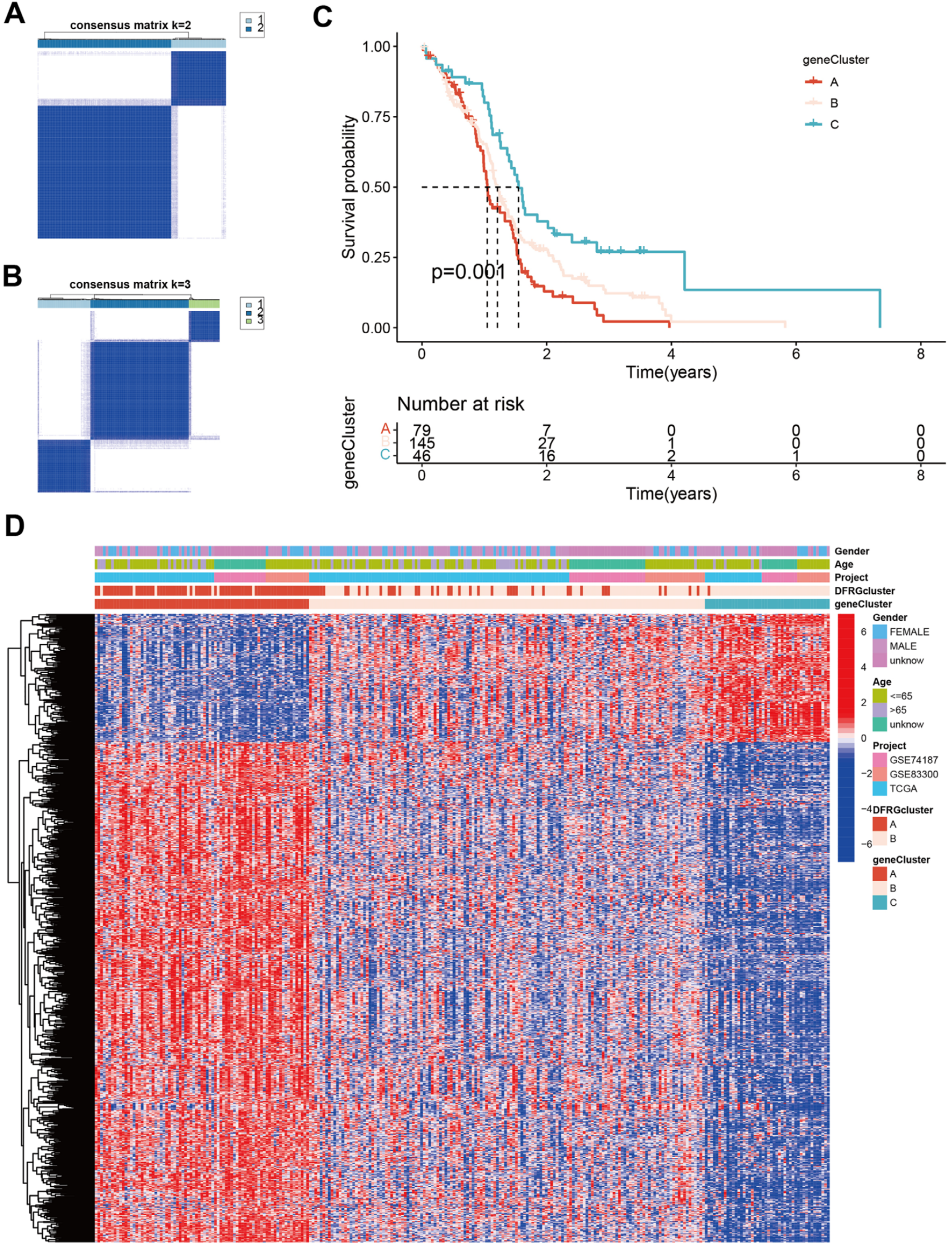
Identification of gene subtypes and comparison of clinical outcomes and characteristics and DFRGs expression levels between the two gene subtypes. **(A)** Unsupervised clustering analysis of prognostic DEGs between the two disulfidptosis subtypes. **(B, A)** Unsupervised clustering analysis of prognostic DEGs between the three disulfidptosis subtypes. **(C)** Kaplan-Meier curves for OS of the three gene subtypes (chi-square test, p = 0.001). **(D)** The heat map shows the differences in clinical features, and DEGs expression levels between geneCluster A, B, and C.

To understand the clinical and molecular heterogeneity within GBM and its implications for patient outcomes, we aimed to identify distinct gene subtypes and compare their clinical characteristics and DEG profiles. Unsupervised clustering analyses were performed to differentiate between disulfidptosis subtypes, as illustrated in **Figures 6A, B**. The identified gene subtypes exhibited significant differences in overall survival (OS), as demonstrated by Kaplan-Meier curves in **Figure 6C**. The chi-square test showed a p-value of 0.001, indicating a statistically significant difference in survival rates among the three gene subtypes. **Figure 6D** features a heatmap that delineates variations in clinical features and **Supplementary Figure S2** shows DEGs expression levels among geneClusters A, B, and C.

### 3.7 Established and validated a risk score model

**Figure 7:**
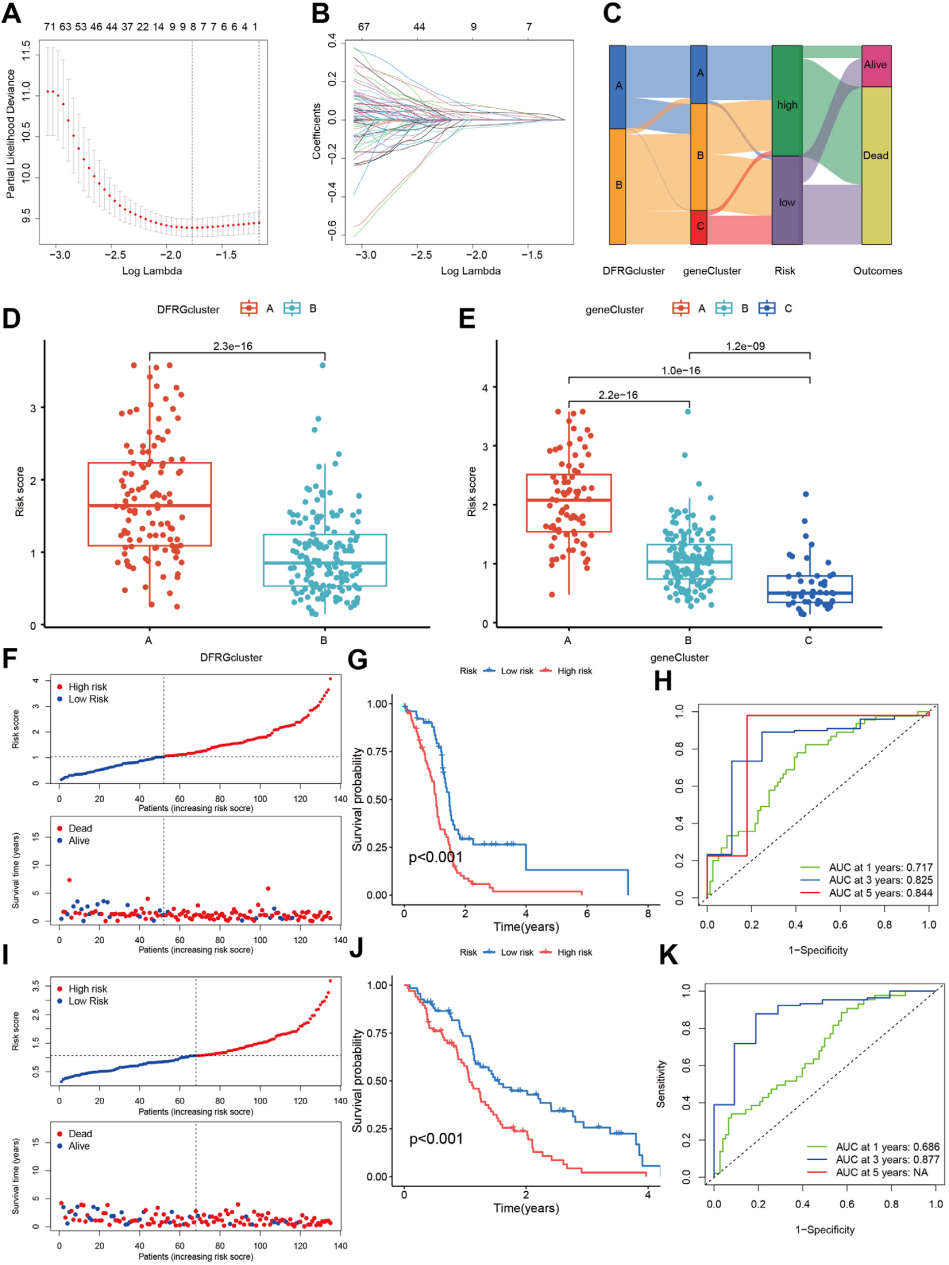
Construction and validation of a risk score model in the training and testing sets. **(A, B)** Utilizing LASSO regression analysis, we identified candidate prognostic genes along with the partial likelihood deviance associated with these prognostic genes. **(C)** An alluvial diagram illustrates the distribution of subtypes and the corresponding prognosis in GBM patients. **(D)** The risk scores demonstrate a distinction between the two DFRG subtypes. **(E)** Similar distinctions in risk scores are observed among the three gene subtypes. **(F)** The risk score for each patient was calculated using the formula: Risk Score = SOD3 × 0.1779 + SPAG4 × 0.2525 + FREM3 × -0.1837 + SPP1 × 0.1810. A ranked dot plot visualizes the distribution of risk scores, while a scatter plot depicts the survival status of patients in the training set. **(G)** Kaplan-Meier curves for overall survival (OS) reveal significant differences between the two risk groups (chi-square test, p< 0.001). **(H)** ROC curves assess the sensitivity and specificity of predicting 0.5-, 1.0-, and 1.5-year survival based on the risk score. **(I)** Another ranked dot plot illustrates risk score distribution, and a corresponding scatter plot depicts patients’ survival status in the validation set. **(J)** Kaplan-Meier curves for OS in the validation set display significant differences between the two risk groups (chi-square test, p< 0.001). **(K)** ROC curves assess the sensitivity and specificity of predicting 0.5-, 1.0-, and 1.5-year survival based on the risk score.

**Figure 8:**
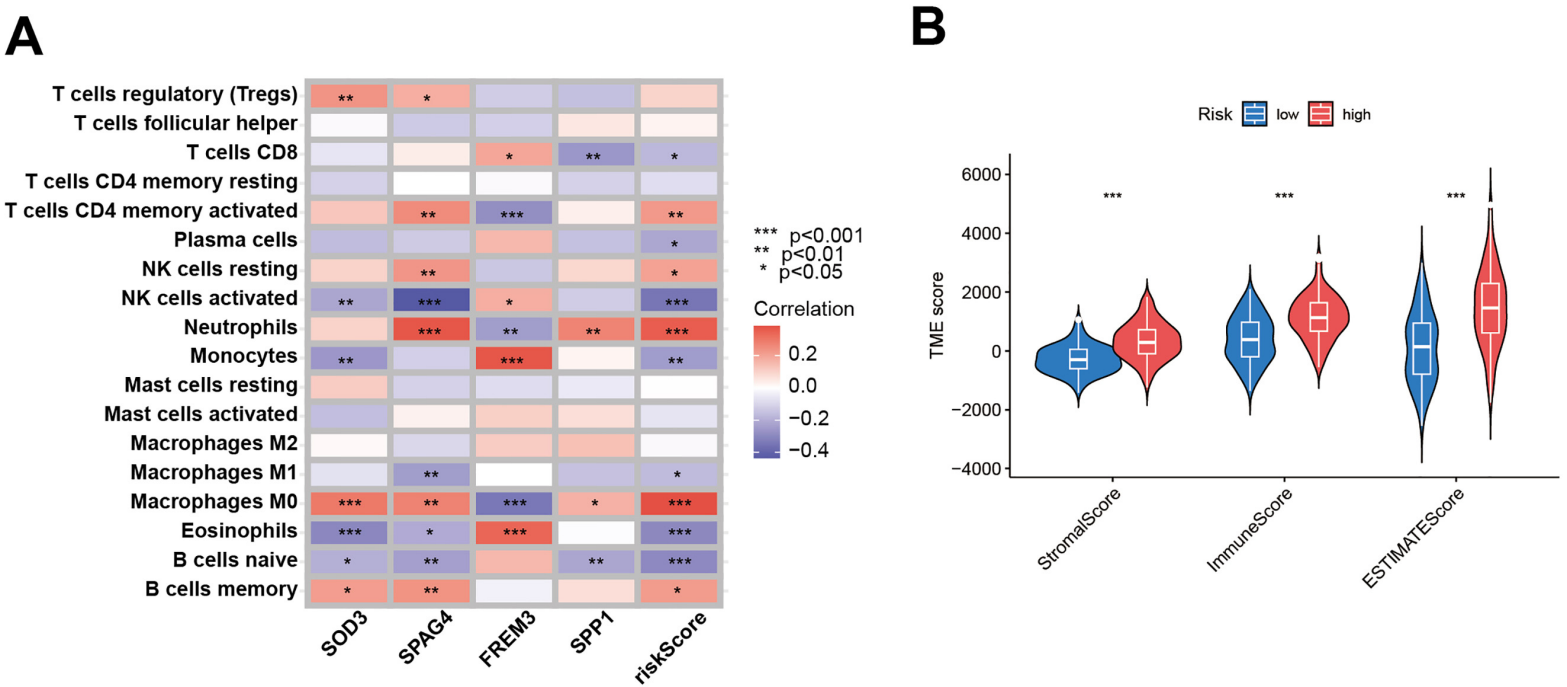
Evaluation of the TME between the two risk score groups. **(A)** Spearman correlation analyses were conducted, and the correlation heatmap illustrates the relationships between the expression levels of 5 model-related genes and the abundance of immune cells. **(B)** The violin plot depicts the three types of Tumor Microenvironment (TME) scores in both the high-risk and low-risk groups.

To discern the prognostic value of DEGs in GBM, LASSO regression analysis was conducted to identify candidate prognostic genes and assessed their association with survival outcomes (**Figures 7A, B**). We also examined the distribution of subtypes and their prognosis in GBM patients, as well as the relationship between gene expression and immune cell abundance (**Figure 7C**). We found that the risk scores, as depicted in **Figure 7D**, effectively distinguished between two DFRG subtypes, and **Figure 7E** showed significant distinctions among three gene subtypes. In the training set, the distribution of these risk scores was visualized in a ranked dot plot (**Figure 7F**). Kaplan-Meier curves in **Figure 7G** revealed significant differences in OS between the two risk groups (chi-square test, p< 0.001), underscoring the predictive power of our risk score model. Furthermore, ROC curves in **Figure 7H** assessed the sensitivity and specificity of predictive model in predicting 1-, 3-, and 5-year survival. The validation set was analyzed similarly, as shown in **Figures 7I–K**. Given the risk model’s proficiency in forecasting the survival rates of GBM patients, we subsequently investigated the correlation between genes associated with the risk model (SOD3, SPAG4, FREM3, and SPP1) and the immune microenvironment. The Spearman correlation analysis, as depicted in the correlation heatmap, indicates a negative correlation between the expression of SPAG4 and the riskScore with the expression of Macrophage M1, suggesting an association with poor prognosis in GBM patients (**Figure 8A**). **Figure 8B** displayed the three types of Tumor Microenvironment (TME) scores in both high-risk and low-risk groups using a violin plot, revealing the immune contexture associated with different risk profiles.

### 3.8 *In vitro* validation of the alleviating effect of SPAG4 on GBM

**Figure 9:**
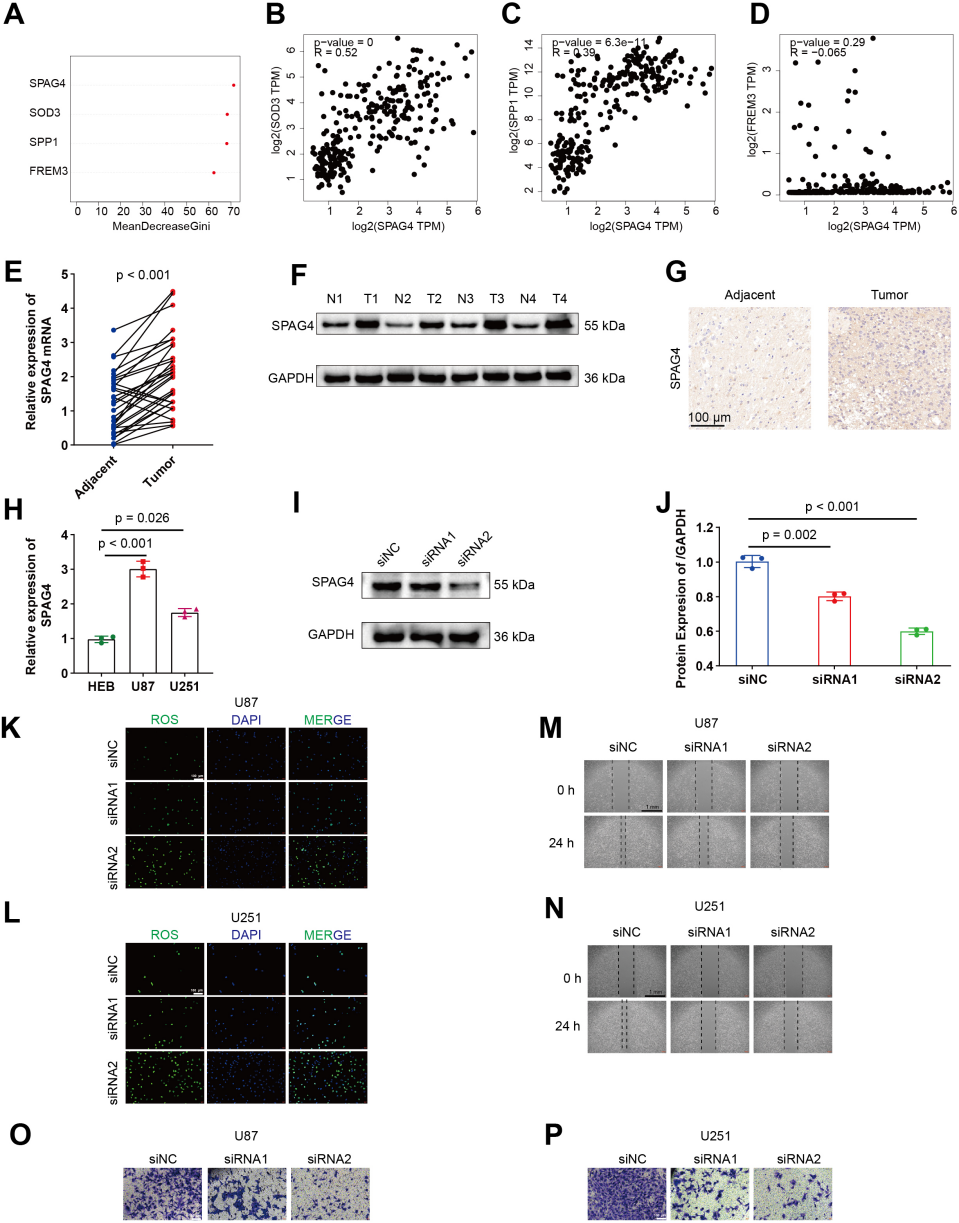
SPAG4 enhances the migratory and invasive capacities of GBM cells by modulating ROS levels. **(A)** Assessment of the relative significance of five model genes. **(B–D)** Spearman correlations between SPAG4 and SOD3, SPP1, and FREM3 using GEPIA. **(E–F)** Comparative expression levels of SPAG4 mRNA and protein in GBM tumor and adjacent normal tissues. **(G)** Representative IHC images illustrating SPAG4 expression in GBM tissues and adjacent normal tissues. **(H)** Relative expression levels of SPAG4 mRNA in HEB, U87, and U251 cell lines. **(I–J)** Immunoblotting validating the efficacy of siRNAs targeting SPAG4 in the U87 cell line. **(K–L)** Depletion of SPAG4 induces an increase in oxidative stress levels in U87 and U251 cell lines. **(M–N)** Wound-healing assays reveal delayed healing in U87 and U251 cell lines upon SPAG4 depletion. **(O–P)** Knockdown of SPAG4 rescues the invasive phenotype of U87 and U251 cell lines.

Firstly, an evaluation of the relative importance of five model genes is presented by random survival forests (RSF) algorithm in **Figure 9A**. Subsequently, Spearman correlations between SPAG4 and SOD3, SPP1, and FREM3 are depicted in **Figures 9B–D**, illustrating their interrelationships using GEPIA. Comparative expression levels of SPAG4 mRNA and protein in GBM tumor and adjacent normal tissues are displayed in **Figures 9E, F**. Representative immunohistochemistry (IHC) images in **Figure 9G** visually capture the differential expression of SPAG4 in GBM tissues compared to adjacent normal tissues. The relative expression levels of SPAG4 mRNA in HEB, U87, and U251 cell lines are presented in **Figure 9H**. Immunoblotting in **Figures 9I, J** effectively confirms the knockdown efficiency of siRNAs targeting SPAG4 in the U87 cell line. Furthermore, depletion of SPAG4 is shown to elevate oxidative stress levels in U87 and U251 cell lines, as evidenced in **Figures 9K, L**. Wound-healing assays in **Figures 9M, N** demonstrate delayed healing in U87 and U251 cell lines upon SPAG4 depletion. Importantly, knockdown of SPAG4 rescues the invasive phenotype of U87 and U251 cell lines, as depicted in **Figures 9O, P**.

### 3.9 SAPG4 facilitates GBM biological function through fat acid metabolism

**Figure 10:**
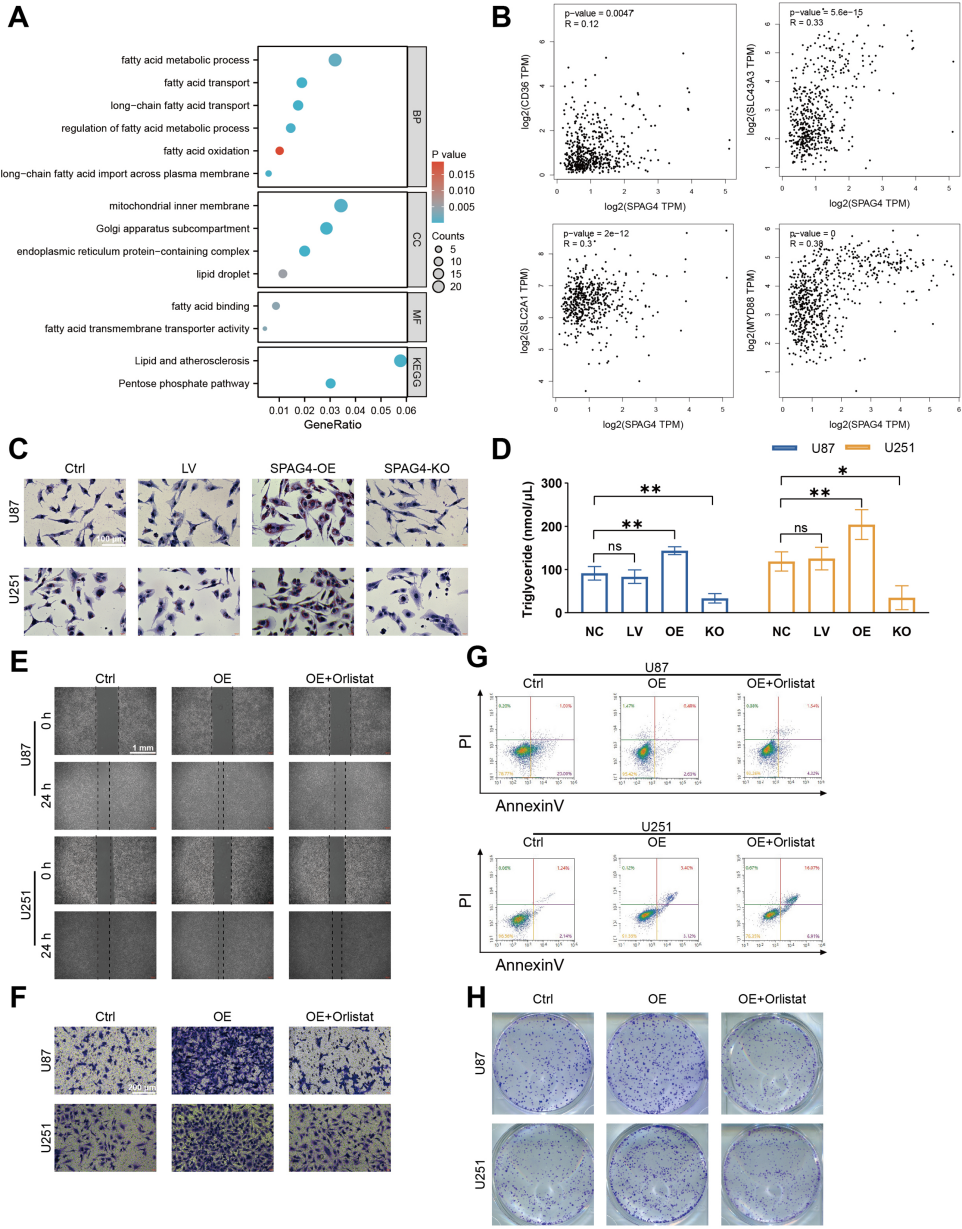
SPAG4 facilitates glioblastoma multiforme (GBM) proliferation, migration, invasion, and apoptosis inhibition through fatty acid metabolism. **(A)** Enriched pathways in GBM cells positive for SPAG4, sourced from the single-cell RNA-sequencing dataset GSE24103. **(B)** Spearman correlation analysis of SPAG4 with CD36, SLC43A3, SLC2A1, and MYD88 using the GEPIA database. **(C)** Oil Red O staining of U87 and U251 cells in control, empty vector lentiviral (LV), SPAG4-overexpressing (OE), and SPAG4-knockout (KO) groups (n=3 per group). **(D)** Measurement of intracellular triglyceride levels in U87 and U251 cells in control, empty vector LV, SPAG4-OE, and SPAG4-knockout groups (n=3 per group). **(E)** Wound healing assay conducted on U87 and U251 cells in control, SPAG4-OE, and SPAG4-OE plus Orlistat-treated groups (n=3 per group). **(F)** Invasion assay performed with U87 and U251 cells in control, SPAG4-OE, and SPAG4-OE plus Orlistat-treated groups (n=3 per group). **(G)** Flow cytometry apoptosis assay on U87 and U251 cells in control, SPAG4-OE, and SPAG4-OE plus Orlistat-treated groups (n=3 per group). **(H)** Clonogenic assay of U87 and U251 cells in control, SPAG4-OE, and SPAG4-OE plus Orlistat-treated groups (n=3 per group). ns means no significance, *P < 0.05 and **P < 0.01.

To delve deeper into the mechanisms by which SPAG4 enhances the proliferative and migratory phenotypes of GBM, we utilized single-cell sequencing data from the GSE24103 dataset, isolating cells with high SPAG4 expression. Subsequent Gene Ontology (GO) and Kyoto Encyclopedia of Genes and Genomes (KEGG) enrichment analyses revealed significant enrichment in biological processes related to fatty acid metabolism (BP), cellular components associated with lipid droplets (CC), and molecular functions involving fatty acid binding (MF). Notably, the KEGG pathway enrichment pointed towards Lipid Metabolism and Atherosclerosis (**Figure 10A**). These findings suggest a correlation between SPAG4 and fatty acid metabolism. To substantiate our hypothesis, we conducted an analysis on GEPIA, examining the correlation between SPAG4 and the significantly enriched genes CD36, SLC43A3, SLC2A1, and MYD88 within the aforementioned pathways. The results indicated a significant positive correlation (**Figure 10B**). Furthermore, Oil-red staining experiments demonstrated that overexpression of SPAG4 significantly increased the content of fatty acids in U87 and U251 cells, whereas SPAG4 knockout markedly decreased fatty acid content (**Figure 10C, D**). Based on these findings, we hypothesize that SPAG4 may modulate the biological functions of GBM by regulating fatty acid metabolism. To test this hypothesis, we treated SPAG4-overexpressing cells with the fatty acid oxidation inhibitor Orlistat. The results showed that the migratory (**Figure 10E**), invasive (**Figure 10F**), anti-apoptotic (**Figure 10G**), and proliferative (**Figure 10H**) capabilities of these cells were significantly affected. This comprehensive analysis provides compelling evidence that SPAG4 could play a pivotal role in GBM biology through its influence on fatty acid metabolism.

### 3.10 *In vivo* validation of SPAG4’s mitigating effect on GBM

**Figure 11:**
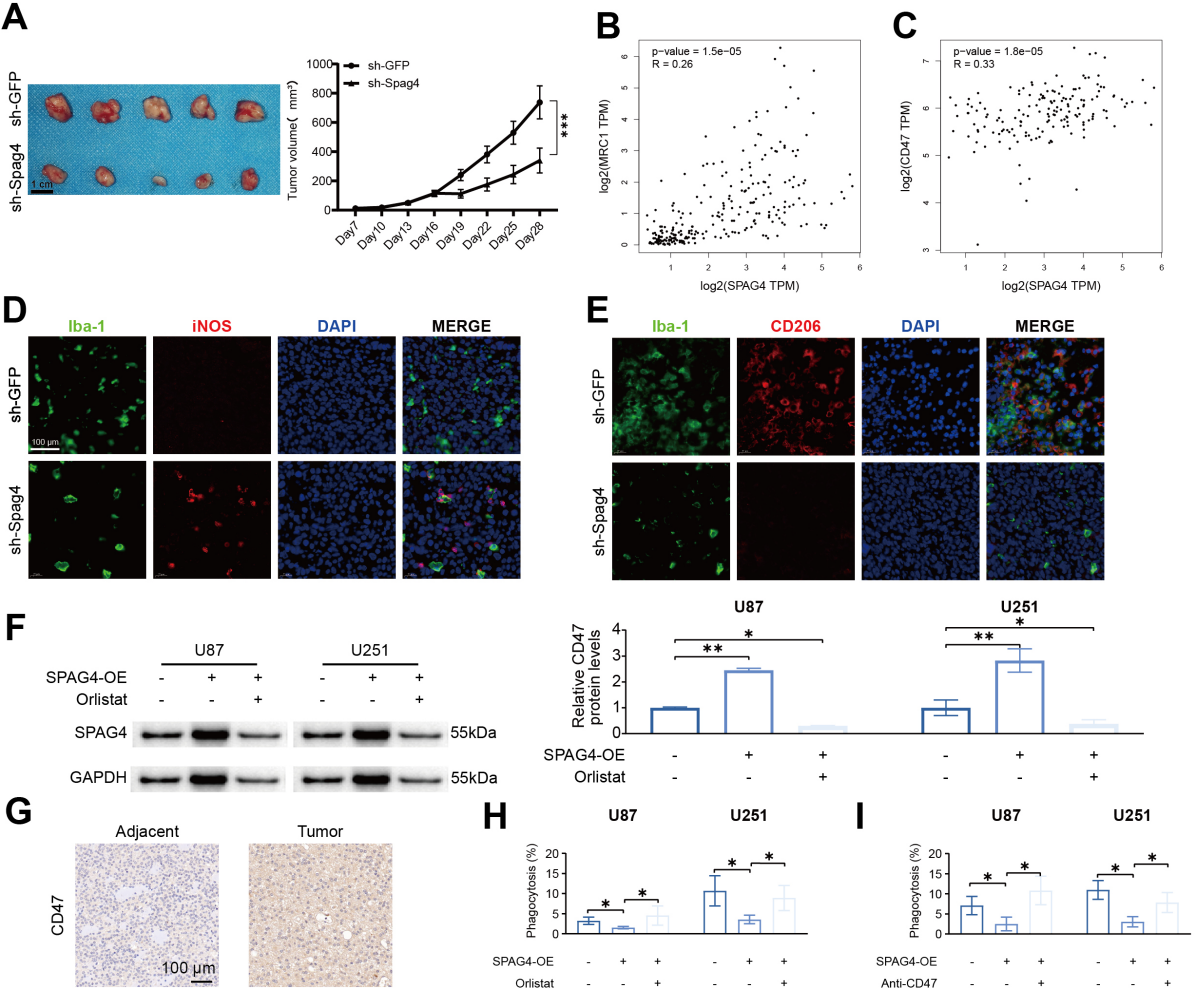
SPAG4 enhances glioblastoma multiforme immune evasion by upregulating CD47 via fatty acid metabolism. **(A)** Subcutaneous xenograft assays were performed utilizing U87-MG cells treated with sh-GFP or sh-Spag4 (the experiment spanned 30 days). Tumor volume following sh-GFP or sh-Spag4 treatment was monitored (n=5 per group). **(B)** Spearman correlation analysis between SPAG4 and MRC1. **(C)** Spearman correlation analysis between SPAG4 and CD47. **(D)** Immunofluorescence staining outcomes for Iba-1/iNOS in subcutaneous xenograft tumors from mice treated with sh-GFP and sh-Spag4. **(E)** Immunofluorescence staining results for Iba-1/CD206 in subcutaneous xenograft tumors from mice treated with sh-GFP and sh-Spag4. **(F)** Western blot (WB) analysis to determine CD47 expression in U87 and U251 cells in control, SPAG4-overexpressing (SPAG4-OE), and SPAG4-OE plus Orlistat-treated groups (n=3 per group). **(G)** Immunohistochemical assessment of CD47 expression levels in glioblastoma and adjacent tissues. **(H)** Phagocytosis assay conducted with U87 and U251 cells in control, SPAG4-OE, and SPAG4-OE plus Orlistat-treated groups (n=3 per group). **(I)** Phagocytosis assay performed with U87 and U251 cells in control, SPAG4-OE, and SPAG4-OE plus Anti-CD47 treated groups (n=3 per group). *P < 0.05 and **P < 0.01.

To evaluate the impact of SPAG4 modulation on U87-MG cell behavior in subcutaneous xenograft assays and to explore its correlation with immune-related markers, we performed subcutaneous xenografts. Our findings revealed significant effects of SPAG4 on the growth and characteristics of U87-MG cells *in vivo*. Notably, a substantial reduction in tumor volume was observed 28 days post-treatment with sh-SPAG4 compared to the sh-GFP group (**Figure 11A**). In further studies to elucidate the role of SPAG4 within the immune microenvironment, we utilized the GEPIA database. Our analysis indicated positive correlations between SPAG4 and MRC1 (R=0.26, p-value=1.5e-5, **Figure 11B**) as well as CD47 (R=0.33, p-value=1.8e-5, **Figure 11C**) in GBM. By downregulating SPAG4 in a mouse GBM xenograft model using sh-Spag4, we observed a significant increase in the M1 macrophage (iNOS) phenotype and a decrease in the M2 macrophage (CD206) phenotype compared to the sh-GFP group (**Figures 11D, E**). Based on these observations, we concluded that SPAG4 likely regulates the immune microenvironment in GBM. Additionally, we hypothesized that SPAG4 might enhance CD47 expression through the promotion of fatty acid oxidation. Overexpression of SPAG4 via lentivirus in combination with the fatty acid synthase inhibitor Orlistat was used to test this hypothesis. The results demonstrated a significant increase in CD47 levels in the SPAG4-OE group, which was abrogated by Orlistat treatment (**Figure 11F**). Furthermore, we investigated whether SPAG4 regulates macrophage phagocytosis via fatty acid metabolism. Our data indicated that the phagocytosis rate in the SPAG4-OE group was significantly reduced compared to the Control group, and Orlistat intervention significantly enhanced the phagocytosis rate in SPAG4-OE (**Figure 11G**). We also explored the possibility that SPAG4 reduces macrophage phagocytosis by upregulating CD47. Our findings showed that the phagocytosis rate in the SPAG4-OE group was significantly decreased compared to the Control group, and treatment with anti-CD47 significantly increased the phagocytosis rate in SPAG4-OE (**Figure 11H, I**). In summary, our research concludes that in GBM, SPAG4 modulates fatty acid metabolism, increases CD47 levels, and consequently evades macrophage phagocytosis, facilitating immune evasion.

## 4 Discussion

Disulfidptosis could instigate a cascade of redox deficiencies and induce cell death. Moreover, the generation of immune responses by immune cells is significantly dependent on the occurrence of redox reactions (7, 8). Interestingly, the redox state constitutes a crucial mechanism in the initiation and progression of tumors (9). Therefore, inducing dual sulfur death holds the potential to be an effective therapeutic strategy for tumors, presenting expansive prospects for research. Extensive research has indicated a close association between the treatment response and prognosis of GBM patients and the infiltration status of immune cells (10–12). However, current research on the association between disulfidptosis and immune-related aspects in GBM is relatively limited. Therefore, this study sought to explore and analyze this relationship utilizing data from TCGA and GEO databases.

Based on the disulfidptosis-related genes (DFRGs) reported by Liu et al., this study selected a subset of 23 DFRGs most strongly associated with disulfidptosis. Among these, 19 DFRGs exhibited mutations, and positive correlations were observed among the DFRGs, suggesting potential cooperative interactions between different DRRGs. Clustering analysis of these DFRGs identified two subtypes, DFRGs A and B, within GBM patients. Notably, the expression of DFRGs was more abundant in the A subtype compared to the B subtype, and patients in the A subtype demonstrated a poorer prognosis than those in the B subtype, suggesting an association between DFRGs and adverse outcomes in GBM patients. Moreover, we conducted a correlation analysis of single-cell sequencing data and observed a significant association between these DFRGS and the progression of GBM. Further analysis revealed higher proportions of immune-infiltrating cells, including CD4+ T cells, dendritic cells, natural killer T cells, regulatory T cells, and helper T cells, in patients with the A subtype of GBM. Previous research has indicated that activated CD8+ T cells contribute to generating a sustained and effective anti-tumor immune response. Dendritic cells and natural killer T cells play crucial surveillance roles *in vivo*, and their dysregulation can facilitate GBM progression. On the other hand, the absence of regulatory T cells and helper T cells may lead to a decline in antigen presentation effectiveness, promoting immune escape. Both the findings from this study and the literature suggest that patients with the A subtype of GBM may have a better prognosis. This implies that these molecular subtypes exhibit distinct immune cell infiltration characteristics and pathways of functional enrichment, and DFRGs may impact the occurrence and development of GBM through mechanisms such as signaling pathway stimulation and regulation of immune cell infiltration in the tumor microenvironment.

SPAG4, a protein associated with sperm function, has recently been implicated in the regulation of Reactive Oxygen Species (ROS) in the context of cancer cell biology (13). According to the literature, SPAG4 interacts with lamin A/C, modulating the nuclear translocation and transcriptional activity of SREBP1, a key regulator of lipogenic pathways. In our study, SPAG4 has been found to enhance the levels of fatty acids in U87 and U251 cell lines. Recent study indicates that fatty acid metabolism in GBM contributes to immune evasion, with one mechanism being the upregulation of CD47 expression (14). Our experimental findings suggest that SPAG4 enhances CD47 expression via fatty acid pathways, thereby reducing macrophage phagocytosis. These results offer novel insights into the immunotherapy of GBM.

In conclusion, Disulfidptosis, as a novel form of cell death, exhibits a close association between its gene expression, the prognosis of GBM patients, and their response to immune therapy. Therefore, targeted therapy focusing on DFRG may offer a novel avenue for diagnosis and treatment in pancreatic cancer. However, limitations such as a relatively small sample size in this study necessitate validation in different cohorts. Furthermore, additional prospective clinical research and fundamental studies are required to further refine and validate these findings.

## Data Availability

The original contributions presented in the study are included in the article/**Supplementary Material**. Further inquiries can be directed to the corresponding author.

## Supplementary material

Supplementary Figure 1
**(A)** 25 disulfidptosis-related candidate genes (DFRGs). **(B)** Utilizing the criteria of |logFC| > 1 and adjusted P.Value < 0.05, we identified a total of 217 upregulated and 149 downregulated differentially expressed genes, encompassing 23 DFRGs.

Supplementary Figure 2 Box plot shows the differences of DFRGs expression levels among geneCluster A, B and C.
