# Supplementary material for: Integrated multi-level omics profiling of disulfidptosis identifis SPAG4 as an innovative immunotherapeutic target in glioblastoma
Source: Front Immunol. 2024 Oct 30;15:1462064. doi: 10.3389/fimmu.2024.1462064 (PMC11557307; doi:10.3389/fimmu.2024.1462064)
Supplement: Supplementary Figure 1 — (A) 25 disulfidptosis-related candidate genes (DFRGs). (B) Utilizing the criteria of |logFC| > 1 and adjusted P.Value < 0.05, we identified a total of 217 upregulated and 149 downregulated differentially expressed genes, encompassing 23 DFRGs. [file DataSheet1.docx]

Integrated multi-level omics profiling of disulfidoptosis identifis SPAG4 as an innovative immunotherapeutic target in glioblastoma

Shenbo Chen^1†^, Man Zeng^2†^, Taixue Chen^1†^, Hui Ding^1^, JiaHan Lin^1^, Fuyue Ye^1^, Ran Wu^1^, Liangwang Yang^1*^, Kun Yang^1*^

^1^Department of Neurosurgery, The First Affiliated Hospital of Hainan Medical University, 31 Longhua Road, Haikou City570100, Hainan Province, China;

^2^Department of Geriatrics Center, The First Affiliated Hospital of Hainan Medical University, 31 Longhua Road, Haikou City570100, Hainan Province, China;

*† These authors contributed equally to this work.*

*** Correspondence:**Kun Yang, chbyk1379@hainmc.edu.cn, Department of Neurosurgery, The First Affiliated Hospital of Hainan Medical University, 31 Longhua Road, Haikou City570100, Hainan Province, China;

Liangwang Yang, 1208353128@hainmc.edu.cn, Department of Neurosurgery, The First Affiliated Hospital of Hainan Medical University, 31 Longhua Road, Haikou City570100, Hainan Province, China;

**
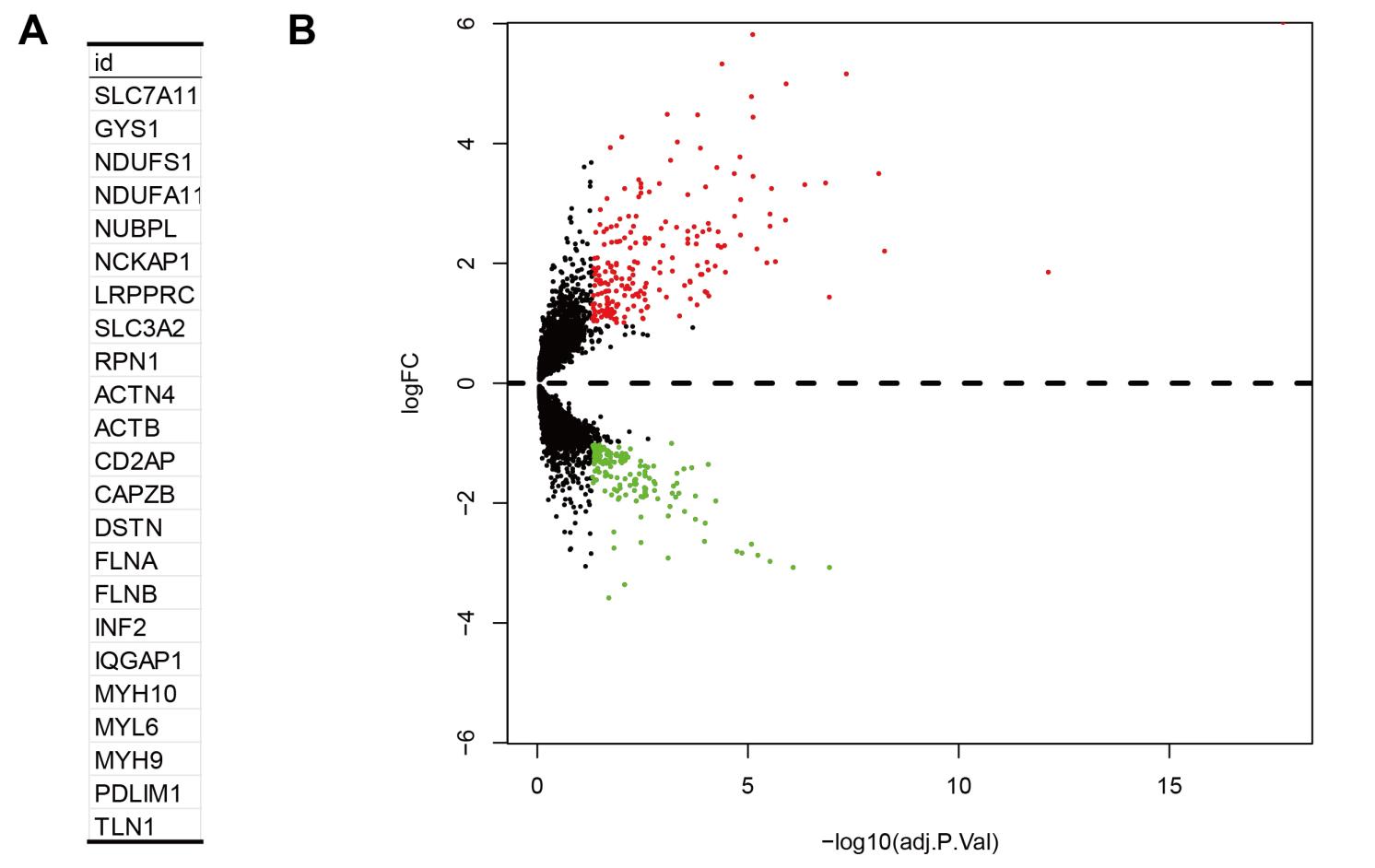
**

**Supplemental Figure 1** (A) 25 disulfidptosis-related candidate genes (DFRGs). (B) Utilizing the criteria of |logFC| > 1 and adjusted P.Value < 0.05, we identified a total of 217 upregulated and 149 downregulated differentially expressed genes, encompassing 23 DFRGs.

**
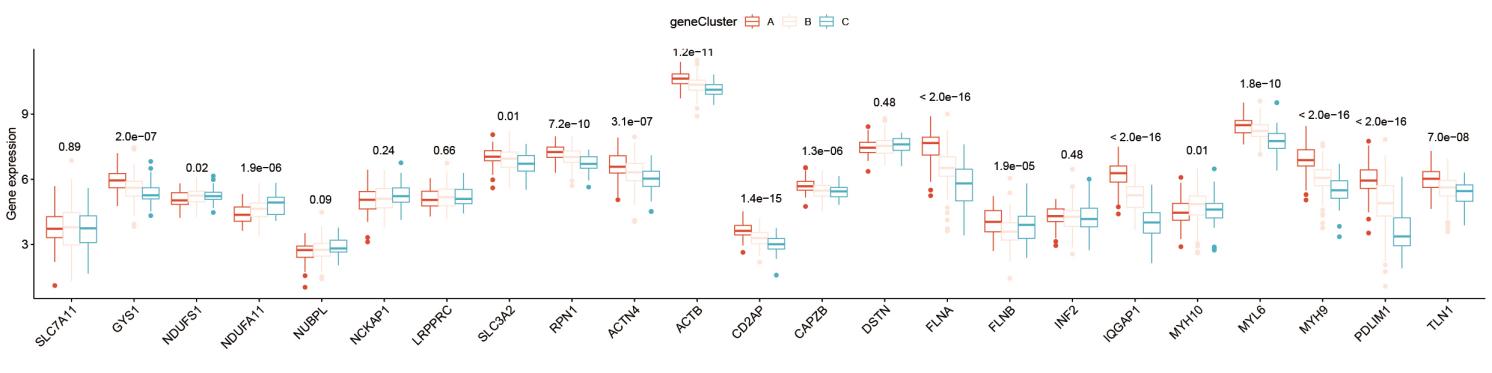
**

**Supplemental Figure 1** Box plot shows the differences of DFRGs expression levels among geneCluster A, B, and C.
